# Performance of HSC Continuous Deep Beams with Asymmetric Circular Openings: Hybrid FRP Versus Steel Plate Strengthening

**DOI:** 10.3390/polym17223049

**Published:** 2025-11-18

**Authors:** Mohammed Al-Mahbashi, Hussein Elsanadedy, Aref Abadel, Husain Abbas, Tarek Almusallam, Yousef Al-Salloum

**Affiliations:** Chair of Research and Studies in Strengthening and Rehabilitation of Structures, Department of Civil Engineering, College of Engineering, King Saud University, P.O. Box 800, Riyadh 11421, Saudi Arabia; helsanadedy@ksu.edu.sa (H.E.); aabadel@ksu.edu.sa (A.A.); habbas@ksu.edu.sa (H.A.); musallam@ksu.edu.sa (T.A.); ysalloum@ksu.edu.sa (Y.A.-S.)

**Keywords:** deep beam, continuous beam, high strength concrete, circular openings, strengthening, FRP, steel plates

## Abstract

In recent years, the demand for high-strength concrete (HSC) for buildings has been steadily increasing. Continuous HSC deep beams are frequently employed in various structural applications, including high-rise buildings, bridges, and parking garages, due to their superior load capacity. Some cases require the addition of openings after the construction for passing utilities such as drainage and electricity. This study experimentally examines four two-span HSC deep beams: one control solid beam, one beam with circular openings, and two beams that utilized different strengthening schemes. The openings were asymmetrical circular openings, with one positioned in each span. This study sought to regain the full capacity of beams with openings by employing two types of strengthening schemes. The first one used bolted steel plates, while the second was a hybrid scheme that combined bolted steel plates with externally bonded fiber-reinforced polymer (FRP) sheets. Test findings demonstrated that both methods effectively restored the load capacity of the strengthened beams. The strengthened beam with steel plates achieved a load capacity of 125% compared to the solid beam. Likewise, the beam retrofitted with hybrid steel/FRP composites reached 117%. Additionally, the energy dissipation and ductility index of the strengthened beam with steel plates were 32% and 77%, respectively, compared to the strengthened beam with hybrid steel/FRP composites. The findings emphasize the effectiveness of the applied retrofitting techniques in restoring the lost capacity due to the cutting of post-construction openings in deep beams.

## 1. Introduction

Beams that have significant depth/span ratios are classified as deep beams. According to ACI 318-25 [1] guidelines, a reinforced concrete (RC) beam is recognized as a deep beam if: (a) *L*/*h* < 4, where *L* is the clear span and *h* is the beam depth, or (b) *a*/*h* < 2, where *a* is the shear span of the beam. In many modern structures, beams are commonly continuous, and recent trends have seen an increased use of high-strength concrete (HSC). In some scenarios, deep beams might require openings to pass utilities, which can adversely affect their shear strength. This effect is particularly pronounced when openings intersect with compression struts, essential components that facilitate load transfer to the supports. These openings can disrupt the load transfer process, compromising the beam’s shear capacity. Moreover, there are situations where openings need to be added to deep beams after construction, potentially diminishing their load-bearing ability. These openings introduce further complexities to the structural performance, particularly when added post-construction. Therefore, strengthening measures become essential to restore and enhance the strength of beams with openings. Steel plates and FRP sheets are among the primary materials used for structural strengthening. While much prior research has focused on the performance and enhancement of deep beams with single spans (e.g., [2,3]) and without openings (e.g., [4,5,6]), studies addressing continuous deep beams and their strengthening remain relatively limited (e.g., [7,8,9]).

Hamoda et al. [10] conducted experimental and numerical studies on RC deep beams having rectangular and circular shapes. All specimens lacked vertical web reinforcement in the strengthening zone to create a defective region. The retrofitting process successfully restored the load capacity of beams with smaller circular openings. In another study [11], researchers utilized FRP composites to enhance the strength of RC deep beams with circular cutouts. The strengthening method using a steel protective frame produced the most significant improvements in load capacity, with increases of 115% over the control specimen with openings and 87% over the control solid beam. Makki et al. [12] studied deep beams made of reactive powder concrete with openings using carbon FRP (CFRP) sheets in different configurations, vertical, inclined, and horizontal. They found that combining inclined strips with anchor bolts enhanced load capacity by 58% compared to the control solid specimen. Rahim et al. [13] tested a single-span RC control deep beam without cutouts and others with cutouts. The CFRP sheets were used for strengthening beams having cutouts. Test results indicated up to 30% reduction in peak load as the size of the openings increased. The improvement in shear strength of beams ranged from 10% to 40%, with three layers being optimal for 200 mm and two layers for 150 mm cutouts.

Allawi et al. [14] tested RC deep beams having square cutouts in the shear span. To strengthen these beams, they applied CFRP sheets. Results revealed an essential decrease of up to 66% in the peak load of beams having openings compared to those without openings. However, the inclusion of CFRP layers increased failure load by 20% to 47% compared to the control beams. Kumari and Nayak [15] investigated 19 beams, one as a reference solid beam and the other beams with square and circular openings. To enhance the beams with openings, they applied glass FRP (GFRP) sheets. The test results revealed a substantial decrease in the load capacity of beams with openings, with a loss of up to 72%. However, the use of externally adhered glass FRP (GFRP) sheets along with gas-actuated fasteners efficiently increased the strength by as much as 64% relative to the non-strengthened specimens. Ten single-span HSC deep beams were tested by El-Mandouh et al. [16]. All the specimens included waste glass powder. Their findings showed that the maximum load and the cracking shear load significantly improved by adding the waste glass powder in beams having openings. Farouk et al. [17] tested twenty RC deep beams with circular and rectangular openings placed in different zones. Of the six beams with rectangular openings, three were strengthened using steel plates of varying thicknesses around the cutouts, while the others were retrofitted with external fasteners. Beams with openings showed about a 71% reduction in failure load compared to solid specimens, and reinforcement in the compression zone had a negligible influence on their behavior. The above studies have shown limitations in fully restoring the capacity of simply supported deep beams. Addressing this gap can lead to improved understanding and repair strategies for continuous deep beams, contributing to safer and more efficient structural designs.

Khalaf et al. [18] investigated strengthened continuous supported RC deep beams having symmetrical square openings using CFRP sheets. The openings were positioned in the middle of spans. Their findings showed that the strength of RC beams with openings reached 79% of the solid specimen, while the retrofitted beams achieved 93%. In a previous study, the authors [19] investigated the effects of asymmetric circular and rectangular openings in high-strength concrete (HSC) continuous deep beams. Two asymmetric openings within the same span reduced the failure load by about 38%, while placing them near the interior and exterior supports of adjacent spans increased the reduction to 45% and 41% for rectangular and circular openings, respectively. Extending this work, Abbas et al. [20] studied the strengthening of HSC continuous deep beams with asymmetric rectangular openings using bolted steel plates. The strengthened beam not only recovered its original capacity but exceeded the control specimen by 9%. They also reported that welding the upper and lower chords to an internal steel box was more effective than increasing plate thickness, and finite element results showed that connecting steel plates around the openings and linking them to the chords enhanced performance, while using only the internal box increased the peak load by 28%.

Previous studies reveal a major gap in understanding the structural response of strengthened continuous RC deep beams having asymmetric openings. This study addresses this gap by focusing on restoring the load-bearing capacity of continuous beams made of HSC that feature two post-construction circular web openings. The openings were strategically situated in the critical shear zones: one disrupting the internal strut of one of the spans and the other intersecting the external strut of the other span. To evaluate the effectiveness of strengthening techniques, four beam specimens were prepared, including one solid control beam without openings and three deep beams containing two circular openings each. The damaged sections were strengthened using two methods: bolted steel plates alone, and a hybrid technique combining bolted steel plates with externally bonded FRP composites. All specimens were subjected to gradually increasing loads applied at the midspan of each span until failure, allowing for a comprehensive assessment of their structural response and load-bearing performance.

## 2. Testing Campaign

### 2.1. Specimen Details

**Figure 1:**
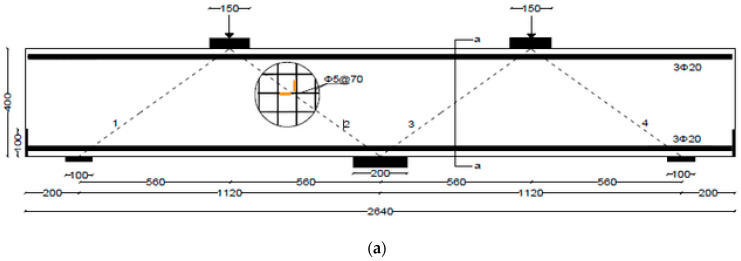

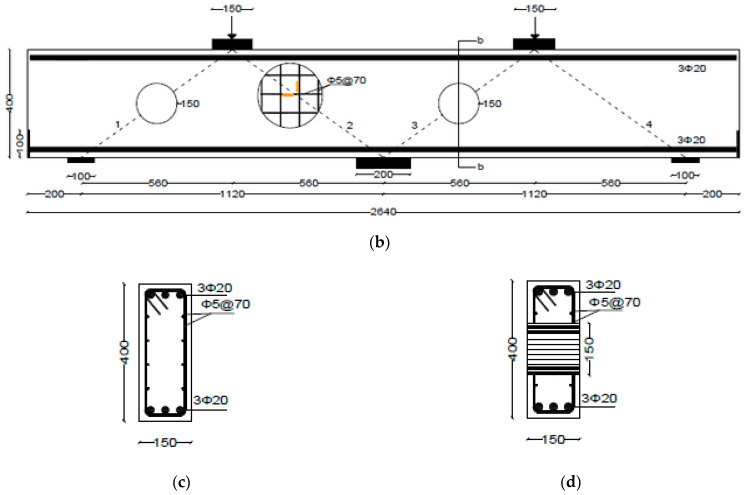
Detailing of rebars and strain gages (brown color) placement in continuous deep beams: (**a**) CS; (**b**) COC13; (**c**,**d**) Secs. a-a, and b-b, respectively (Unit: mm).

The test matrix comprised evaluating four RC two-span deep beams, as shown in Table 1. The cross-section of all beams was 150 mm × 400 mm. The beam’s length was 2.64 m, with effective spans of 1.12 m each, as illustrated in Figure 1. The solid control beam, designated as CS, served as the first specimen and did not contain openings. The other three beams featured two circular cutouts, each of 150 mm diameter (equivalent to 38% of the beam depth). These openings were asymmetrically positioned at the midpoint of the imaginary lines connecting the centers of the support and loading plates (Figure 1). This opening layout was selected, as it represents a unique case in which asymmetrical openings are positioned to cut the load path in both the interior and exterior main diagonal strut simultaneously. The beams with openings were strengthened using two different schemes. The circular openings in the beams were pre-constructed, as they were created during the formwork stage before casting, ensuring accurate placement of openings. The first scheme (S1) utilized externally bonded steel plates, while the second scheme (S2) employed a hybrid system combining FRP composites with bolted steel plates. All beams were reinforced with 3Φ20 mm deformed steel bars at both the bottom and top. The rebars in the web were in the form of a mesh of Φ5 mm steel bars spaced equally at 70 mm in both horizontal and vertical directions, conforming to the minimum reinforcement ratio and maximum spacing requirements specified in ACI 318-25 [1]. Detailed reinforcement arrangements are presented in Figure 1.

Steel plates were used at all supports and loading locations to minimize stress concentrations and prevent premature deterioration in the concrete. These plates were extended across the full width of the beams to ensure uniform load distribution. At the external supports, 20 mm thick plates were used, while 40 mm thick plates were provided at the loading locations and interior support to accommodate higher stress demands. All tested specimens were tested under quasi-static loads acting at the middle of each span.

### 2.2. Characteristics of Materials

The RC continuous deep beams were constructed using ready-mixed concrete designed for a compressive strength of 60 MPa. At 28 days, the actual compressive strength, determined through standard cylinder testing as per ASTM C39/C39M [21], was recorded as 63 MPa. The mechanical properties of the reinforcing steel were established through direct tension testing conducted as per ASTM E8/E8M [22]. The main longitudinal reinforcement, consisting of Φ20 mm, exhibited an ultimate tensile strength of 697 MPa, and a yield strength of 554 MPa, and an elongation at fracture of 11.3%. The 5 mm diameter bars used as web reinforcement had an ultimate strength of 550 MPa and a yield strength of 527 MPa. High-strength steel bolts, used to secure steel plates to the RC beam, had an ultimate strength of 1345 MPa and a yield strength of 711 MPa. The steel plates used for beam strengthening possessed a yield strength of 230 MPa and an ultimate strength of 345 MPa. The mechanical properties of unidirectional CFRP and GFRP laminates obtained from the tests include the elastic modulus of 71 GPa and 20.9 GPa, ultimate strength of 710 MPa and 253 MPa, and ultimate strain of 1% and 1.2%, respectively. The CFRP system had a layer thickness of 0.6 mm, while the GFRP system had a thickness of 1.3 mm per layer.

### 2.3. Strengthening Schemes

The primary goal of strengthening the beams having openings was to recover the ultimate strength of the beams. Two schemes were used: the first scheme (S1) involved the use of bolted steel plates, whereas the second scheme (S2) employed bolted steel plates combined with externally bonded FRP composites (hybrid system). The fundamental concept behind these schemes was to strengthen the regions surrounding the openings.

**Figure 2:**
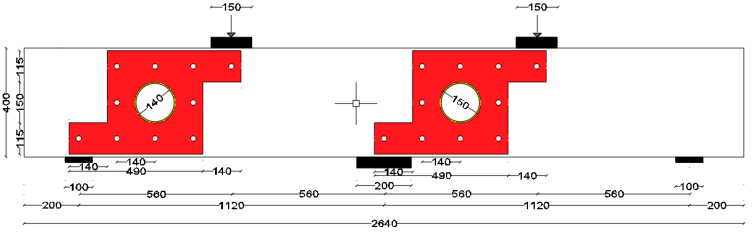
Layout of the first strengthening scheme adopted for beam COC13S1 (Unit: mm).

**Figure 3:**
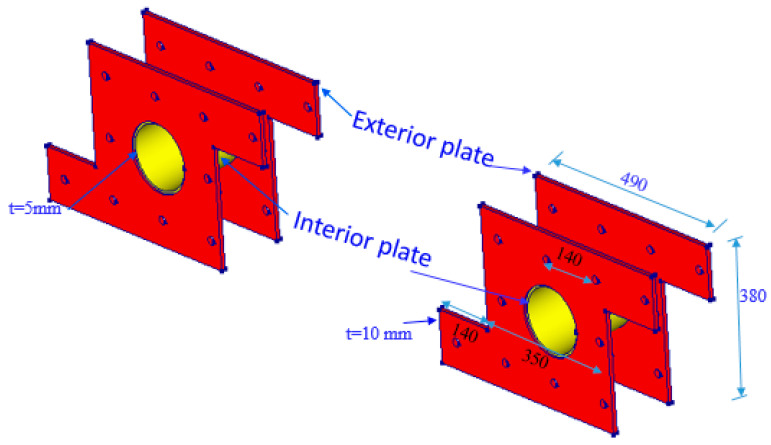
Steel plates used in first strengthening scheme adopted for beam having openings (Unit: mm).

The first strengthening scheme involved the installation of steel plates around the openings, as depicted in Figure 2. This method was selected to facilitate the effective transfer and distribution of loads from the load to the support points, which was compromised by openings. Anchor bolts were used to securely affix a custom-shaped steel plate (shown in Figure 3) to both sides of the beam. The external steel plates were welded with a 5 mm thick internal steel cylinder inserted in the opening. The design of the bolts adhered to the AISC 360-16 provisions [23]. The spacing between the bolts, both horizontally and vertically, was set at 140 mm, which is twice the distance between the web rebars.

**Figure 4:**
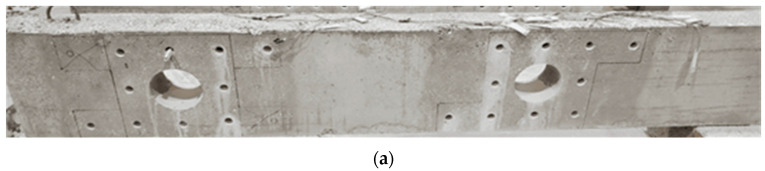

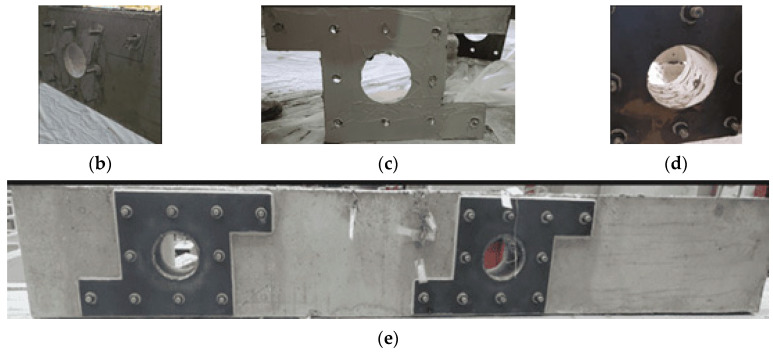
Procedure of strengthening beam using the first scheme (i.e., using steel plates): (**a**) drilling holes for anchor bolts; (**b**) fixing threaded rods in holes with epoxy; (**c**) epoxy applied to steel plates; (**d**) fixing exterior plates, tighten the anchors and apply epoxy on inside surface of opening; and (**e**) fix the steel pipe of interior plates and weld it with outside plates.

Figure 4 outlines the steps for implementing the first scheme, S1. Prior to attaching the steel plates, the concrete surfaces near the openings were prepared by sandblasting. ASTM A36 [24] grade steel plates, each 10 mm thick, were attached to the outer faces of the concrete deep beam surrounding the openings. Holes were drilled through the width of the beam, allowing 18 mm high-strength threaded steel rods to be inserted through these holes. Epoxy adhesive mortar (Sika-300, Sika Saudi Arabia Limited, Jeddah, Saudi Arabia) was also employed to secure the steel plates to the concrete. Pressure was applied to expel any epoxy that squeezed out from between the concrete surface and the steel plate. Nuts were threaded onto the rods to securely tighten the steel plates to the concrete surface. Epoxy bonding mortar was employed to fill the gaps between the threaded rods and the holes. The process of strengthening is illustrated in Figure 4.

**Figure 5:**
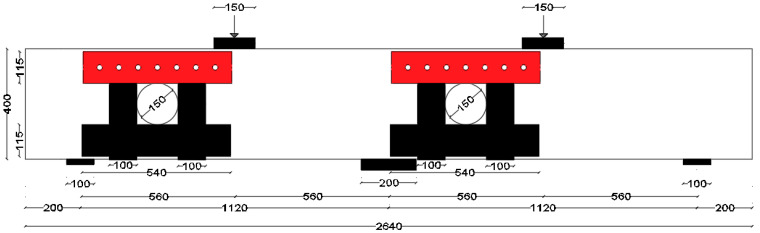
Layout of the second strengthening scheme adopted for beam COC13S2 (Unit: mm).

The second scheme of strengthening involved fixing unidirectional FRP sheets around the openings with top chord steel plate, as shown in Figure 5. The bottom horizontal layer provided below the openings had four layers of CFRP sheets with fibers oriented parallel to the span of the beam, whereas the top horizontal layer had three layers of CFRP sheets overlaid by one layer of GFRP sheet with fibers oriented parallel to the span of the beam. GFRP was employed underneath the steel plates to prevent galvanic corrosion at the CFRP/steel interface. U-wraps of three layers of CFRP sheets, in addition to one layer of GFRP sheet with fibers oriented vertically, were added around the openings on top of the horizontal FRP layers. For anchoring the top ends of U-wraps of FRP, a 5 mm thick steel plate was attached to the top chord of the beam above openings. To avoid galvanic corrosion of the steel plate, the contact between the CFRP sheets and the steel plate was avoided by providing a layer of GFRP sheet. It is due to this reason that instead of providing four layers of CFRP sheets in all, the upper layer (above the opening) and U-wraps, which were in contact with the steel plate, were made of GFRP sheets.

**Figure 6:**
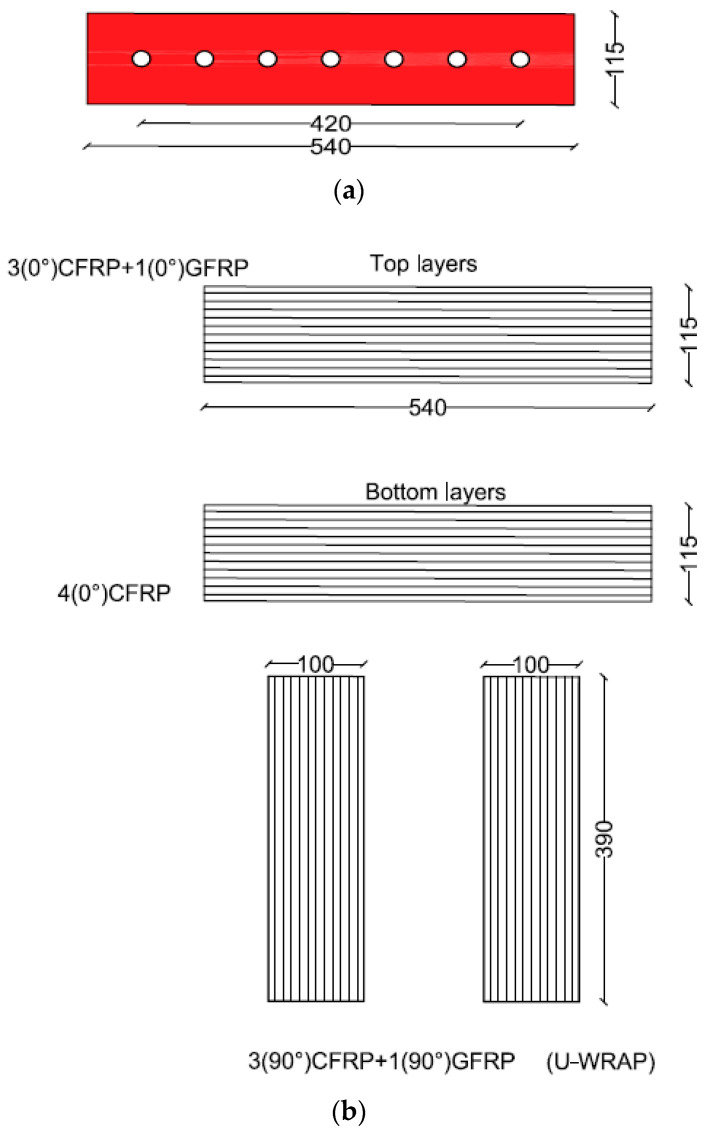
(**a**) Size of steel plate and the location of anchors; (**b**) Sizes and number of layers of FRP sheets used in the top and bottom layers and U-wraps (Unit: mm).

The top and bottom horizontal layers were 115 mm wide and 540 mm long, while the U-wrapped layers were 100 mm wide, covering the full depth of the beam, as illustrated in Figure 6. The anchors, used for attaching the steel plate to the beam, had a center/center distance of 70 mm.

**Figure 7:**
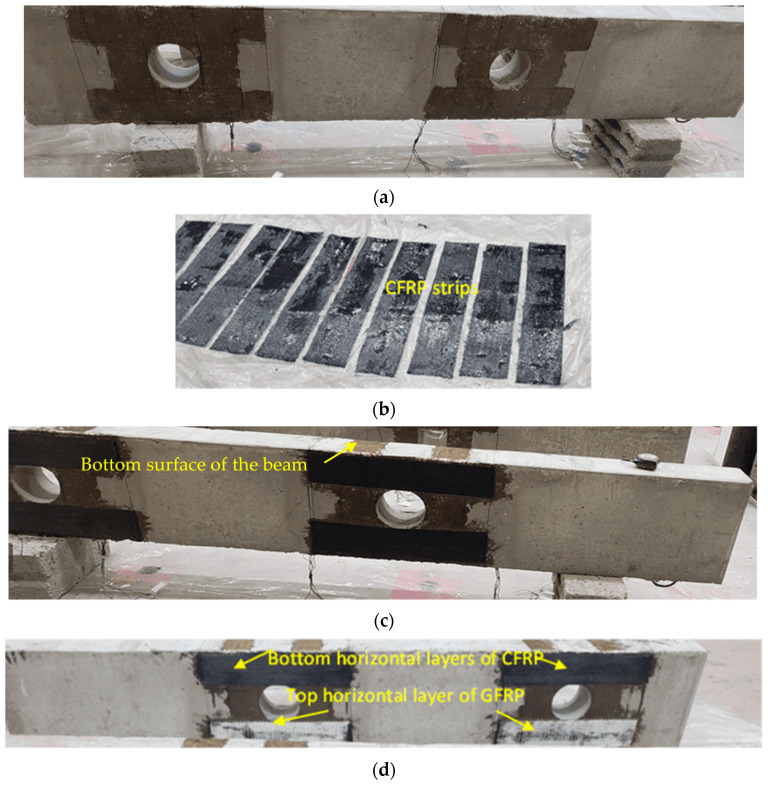

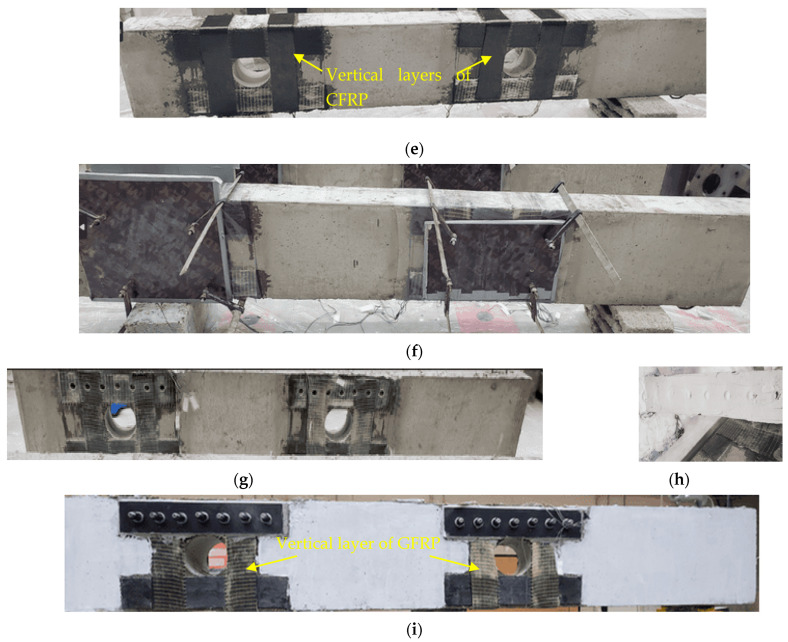
Fabrication of strengthened specimens using FRP composite materials (second scheme): (**a**) epoxy painting of concrete and FRP; (**b**–**e**) fix CFRP and GFRP sheets on beam; (**f**) fixing wooden frame for keeping the FRP sheets pressed; (**g**) drilling holes for anchors; (**h**) painting the surface of steel plates with epoxy; and (**i**) fix steel plates, pass anchor rods through holes and tighten bolt.

Figure 7 illustrates the strengthening strategy for the second scheme (S2). The wet layup method was utilized for attaching the FRP sheets to concrete. The holes were drilled at the locations of the anchor bolts. The concrete surface was cleaned, dried, and made free from any loose particles, oil, or grease. It was also roughened by sandblasting to improve adhesion. A thin layer of the epoxy resin Sika-300 was spread on the prepared surface of the RC using a brush. The entire area to be covered by the FRP sheet was evenly coated with epoxy resin. The fiber sheets were joined together by epoxy before being applied to the adhesive-coated concrete surface. The FRP sheets were carefully placed onto the wet resin-coated concrete surface, ensuring good contact between the FRP and the concrete. A roller was used to roll out any air bubbles or wrinkles in the FRP sheet for ensuring a smooth and even bond between the FRP layers and the RC. The wooden boards were used for keeping the FRP layers pressed to ensure the adhesion of the fiber layers together and with the concrete surface. The resin was allowed to cure and bond the FRP to the concrete, and then the wooden boards were removed. Subsequently, the steel plates were placed in the top chord, and threaded rods were used to securely anchor them to the concrete surface by tightening nuts.

### 2.4. Instrumentation and Experimental Setup

**Figure 8:**
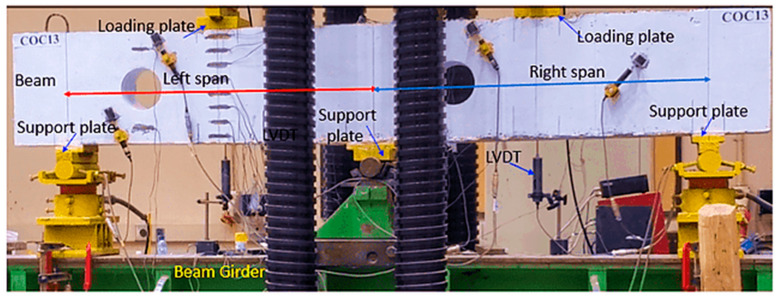
Continuous beams testing setup.

To measure the vertical displacement of the center of each span of the beam, two linear variable displacement transducers were utilized on the bottom surface of the beam. To capture strain data, strain gages were installed on the main longitudinal rebars at the midpoints of the spans and on the top bars at the interior support. Additionally, two strain gages were positioned on the horizontal and vertical web rebars, aligned with the midpoint of the diagonal strut (Figure 1). An AMSLER test machine (Alfred J. Amsler & Co., Schaffhausen, Switzerland) was used for testing both unstrengthened and strengthened beams. Test specimens were subjected to quasi-static point loads applied at mid-span at a rate of 0.5 mm/min under a displacement-controlled strategy. Load, deflection, and strain data were recorded at 1 Hz using a data acquisition system. Loading was continued monotonically until about a 50% drop in peak load, capturing post-peak behavior while avoiding sudden failure. Short dwell periods were intermittently introduced to mark and document newly formed or propagating cracks. The setup for testing the beam with openings is depicted in Figure 8.

## 3. Analysis and Discussion of Results

### 3.1. Failure Types and Cracks’ Development

In the control solid deep beam, diagonal cracks initiated near the middle support, propagating toward the loading plate positioned in the right span.

**Figure 9:**
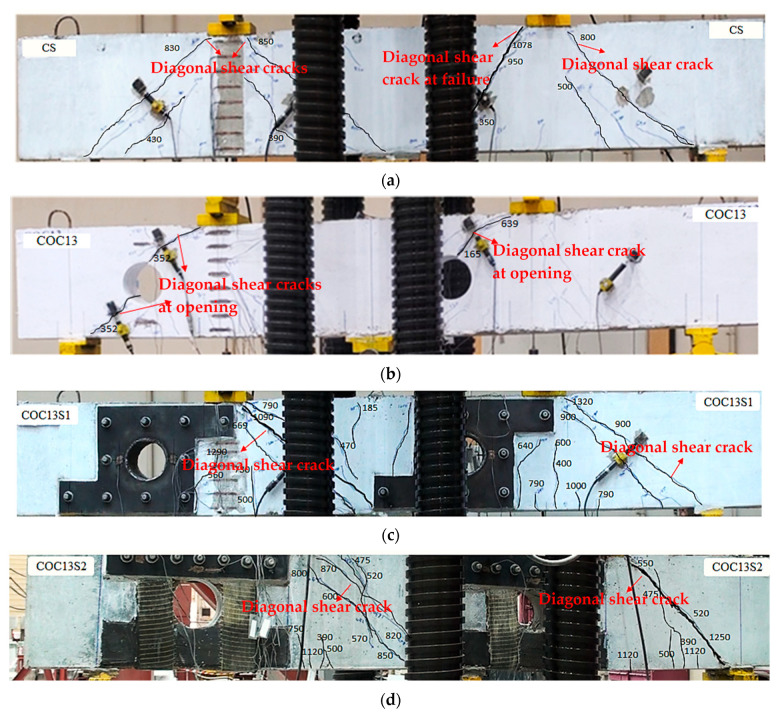
Peak load crack patterns in test specimens: (**a**) CS; (**b**) COC13; (**c**) COC13S1; and (**d**) COC13S2.

**Figure 10:**
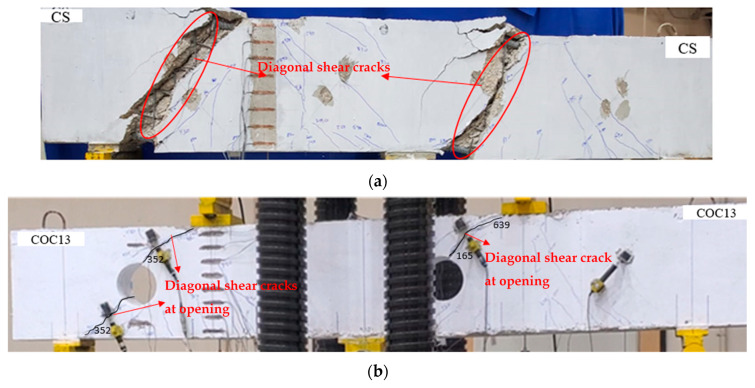

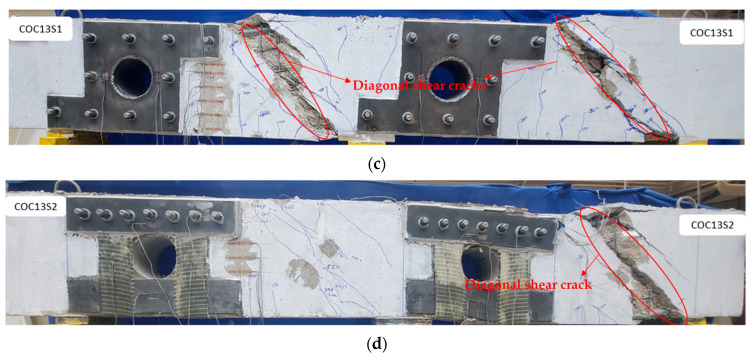
Testing specimen’s final failure: (**a**) CS; (**b**) COC13; (**c**) COC13S1; and (**d**) COC13S2.

In the control solid deep beam, diagonal cracks initiated close to the middle support in the direction of the loading plate, which was located in the right span when a load of 350 kN (0.32$P_{u}$, where $P_{u}$ is the peak load) was applied. An additional inclined crack initiated in the left span at the exterior support as the load increased. Over time, the diagonal cracks propagated towards the corner of the loading plates. The beam ultimately failed at an ultimate load of 1078 kN (=$P_{u}$), as illustrated in Figure 9a. Following this failure, there was a slow decrease in the load capacity, and the cracks expanded until final failure occurred. The beam was subjected to loading until there was approximately a 50% reduction in peak load, which resulted in the crushing of struts, as shown in Figure 10a.

The cracks began developing between the top chord of the interior opening and the load point in the right span at a load of 165 kN (0.26$P_{u}$) for beam COC13. When the load reached 352 kN (0.55$P_{u}$), additional cracks appeared between the opening and the exterior support, as seen in Figure 9b. The beam ultimately failed at 639 kN with the final failure of COC13 shown in Figure 10b. As deep beams primarily fail through strut splitting, leading to diagonal shear cracks, the introduction of openings weakened the strut in that region, resulting in a lower ultimate load. However, since the failure mechanism remained governed by strut crushing and splitting, the crack pattern appeared similar to that of the control beam, as seen in Figure 10b.

The first crack in the COC13S1 beam, strengthened using steel plates (scheme S1), developed at about 400 kN and 500 kN (0.30$P_{u}$ and 0.37$P_{u}$) in the flexural regions for the right span and left span, respectively. This indicates that the first crack load of COC13S1 is higher than the control solid beam CS (350 kN). The enhancement in the first crack load can be ascribed to the use of stiff steel plates. Other vertical flexural cracks developed at the edges of steel plates at 560 kN and 640 kN (0.42$P_{u}$ and 0.48$P_{u}$). The diagonal shear crack started developing in the right and left spans at 415 kN and 480 kN (0.31$P_{u}$ and 0.36$P_{u}$), respectively. The progression of cracks increased in the vertical and inclined direction until reaching an ultimate load of 1344 kN (Figure 9c). The loading on the beam continued until final failure, as shown in Figure 10c.

The flexural cracks started developing in the COC13S2 beam, strengthened using scheme S2, between 390 kN and 500 kN (0.31–0.40$P_{u}$) for the two spans. At a load of 475 kN (0.38$P_{u}$), inclined shear cracks formed in both spans. The cracks continued to widen and extend between the supports and loading plates until reaching up to 1263 kN, as shown in Figure 9d. The loading was sustained until the final strut failure, as shown in Figure 10d.

It is worth mentioning that the first crack load is not reported for the strengthened specimens, as the occurrence of the first crack could not be determined with certainty. The cracks may have initiated either within the areas covered by the strengthening materials or in the uncovered regions. Since the externally bonded layers concealed direct visual observation, reporting the first crack load based solely on the uncovered portion would not accurately reflect the actual cracking behavior of the strengthened beams.

It is worth noting that the observed failures were confined to the concrete portions of the beams, while the reinforcing materials remained intact throughout the loading process. No debonding was detected at the steel plate–concrete interface, and the FRP reinforcement exhibited neither fiber breakage nor U-shaped stirrup anchorage failure. The reinforcement interfaces maintained full bond until and after the ultimate load was reached. Failure was primarily governed by concrete cracking and crushing, as illustrated in Figure 10.

It is worth mentioning that full coverage around the opening was not feasible with the transverse layout of unidirectional FRP sheets in the second strengthening scheme (COC13S2), as seen from Figure 5. Adding diagonal layers would still leave minor uncovered areas and lead to excessive FRP use. However, the surrounding region was adequately strengthened, preventing failure near the opening, as evident in Figure 10d. Future studies may consider using bidirectional FRP sheets to achieve complete coverage around the opening.

### 3.2. Load-Deflection Response

**Figure 11:**
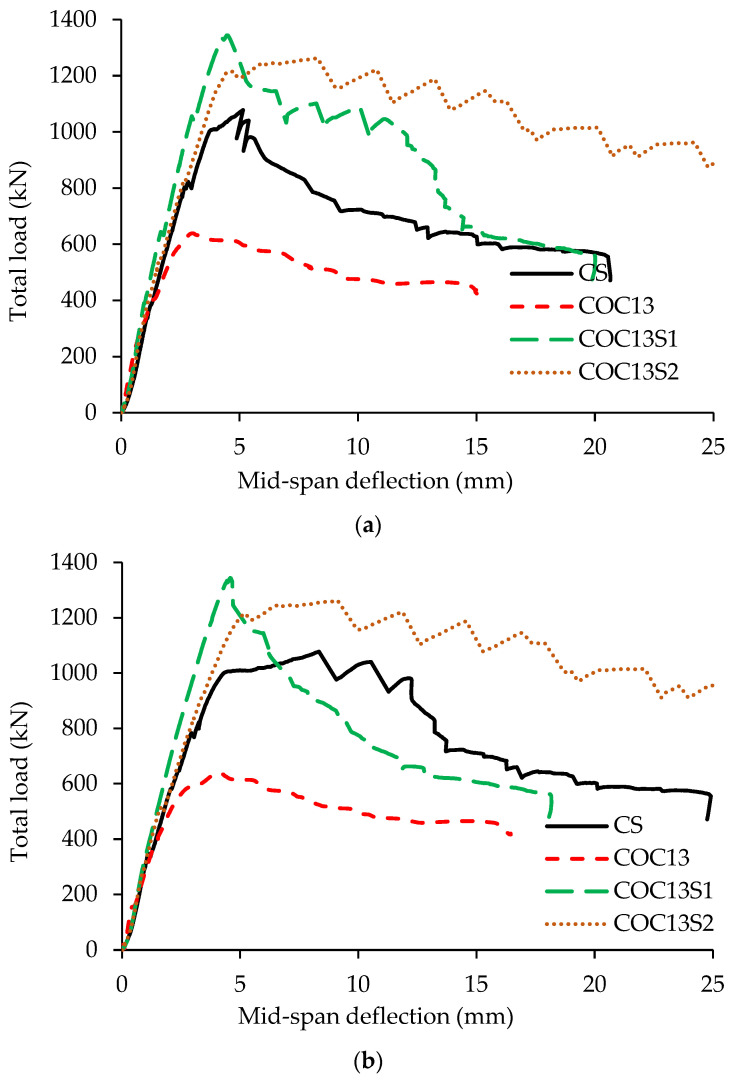
Load-deflection variation for test specimens of COC13S1 and COC13S2: (**a**) left span; and (**b**) right span.

Figure 11 compares the load versus mid-span deflection plots of strengthened beams having openings (COC13S1 and COC13S2) with the control (CS) and the beam having openings before strengthening (COC13).

The key parameters derived from the load-deflection response of strengthened RC beams are listed in Table 2. These include the peak load, right and left span deflections at peak load, deflections at the ultimate stage, and energy absorption up to the ultimate stage. The ultimate stage corresponds to a 20% drop in peak load, as per NZS [25].

**Figure 12:**
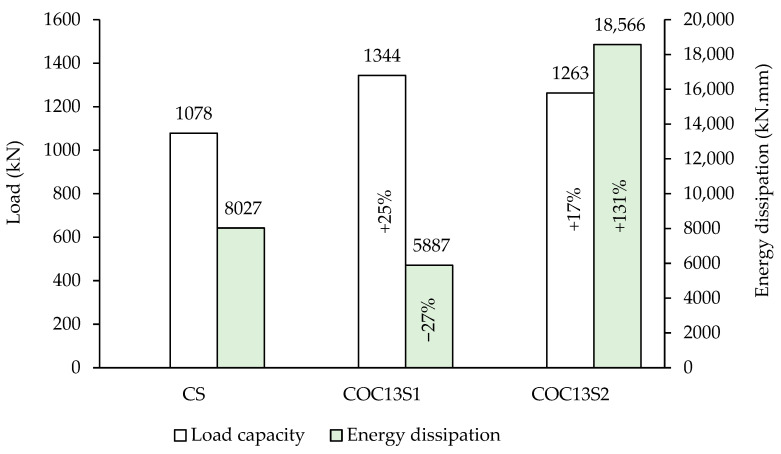
Load capacity and energy dissipation in tested beams.

Figure 12 presents a bar chart comparison of the peak loads for all tested beams. The COC13 beam (with openings but without strengthening) is not included in this figure because comparing it with other beams (CS, COC13S1, and COC13S2) in terms of energy dissipation or ductility (later in Section 3.4) would be inappropriate, as it would fail prematurely and would not represent meaningful performance. It is evident that the introduction of two circular openings in specimen COC13, one located in the right span crossing the interior strut and the other in the left span near the exterior support, led to a 41% reduction in load-bearing capacity. This significant decrease can be attributed to the disruption of the primary strut paths between the supports and loading points. Upon strengthening, beam COC13S1 exhibited a notable recovery, with its peak load increasing by 110%, outperforming the solid control beam CS by 25%. Similarly, the strengthened specimen COC13S2 achieved a 98% improvement, exceeding the capacity of the control beam CS by 17%. These findings highlight the significant effectiveness of the adopted strengthening techniques. These results emphasize the effectiveness of the applied strengthening schemes. The higher strength observed in COC13S1 indicates that the steel plate configuration provided greater confinement by covering the whole area around openings and was more conservative than the FRP scheme, although both showed improved performance over the control beam. Thus, the actual strengthening requirement is quite less, with COC13S1 providing more excess reinforcement than COC13S2.

The deflections of the two spans for beams with openings show greater deflection in the right span compared to the left one. This may be due to the weakness of the right span, as the opening interrupts a relatively more critical load path line connecting the interior support with the loading plate. The average deflection at peak load for CS was measured at 6.7 mm. However, the deflection at peak load of the strengthened beam COC13S1 got reduced to 4.5 mm, which is 68% of CS. For the second scheme, beam COC13S2, the left span deflection at peak load increased to 8.3 mm, which is 23% more than CS.

At the peak load, beam COC13 exhibited mid-span deflections of 4.1 mm and 3.0 mm in the right and left spans, respectively. For beam COC13S1, strengthened using the first scheme, the corresponding deflections increased slightly to 4.6 mm and 4.5 mm. However, at the peak load, the deflections of beam COC13S2, strengthened using the second scheme, were significantly higher at 9.1 mm (right span) and 8.3 mm (left span). A comparison of the two strengthening schemes reveals that COC13S1 achieved a 6% increase in the load capacity along with a substantial reduction in deflection, approximately 50% in the right span and 54% in the left span, relative to COC13S2, as detailed in Table 2. Moreover, the measured span-to-deflection ratio of the beams at working load exceeded 700, indicating that the deflections were small (less than 1.58 mm) and well within typical serviceability limits.

For the strengthened specimens (COC13S1 and COC13S2), the response of both spans was nearly symmetrical, as the applied strengthening effectively compensated for the weakness in the compression struts caused by the openings. This is also reflected in the comparable deflection profiles of the two spans. In contrast, the unstrengthened beam (COC13) exhibited higher deflection and earlier failure in the right span containing the inner-strut opening, indicating that failure of the inner compression arch has a more pronounced influence on the overall load-bearing capacity. However, the behavior of the unstrengthened beam is of limited practical relevance, as such beams would typically be strengthened in real applications.

However, the average mid-span deflection of the strengthened beam using steel plates (COC13S1) was almost 52% of the deflection of the beam strengthened using FRP (COC13S2). A comparison with the control beam (no openings) shows that the average mid-span deflection of the beam strengthened using steel plates (COC13S1) is 67% of the control, whereas for the beam strengthened using FRP (COC13S2), it is 31% more than the control. A sudden drop in deflection was observed for the strengthened beam (COC13S1) after reaching the peak load, indicating a brittle failure behavior. In contrast, the COC13S2 beam, which was strengthened using fiber materials (FRP composite), exhibited a more gradual reduction in deflection beyond the peak load. This behavior reflects a more ductile response and demonstrates improved post-peak ductility of the COC13S2 beam compared to the COC13S1 beam. The higher load capacity and smaller deflections in COC13S1 are attributed to the use of stiffer strengthening material (steel plate) in this specimen as compared to the flexible (FRP composite) materials adopted in COC13S2.

The energy dissipation in the beam using FRP composite materials (COC13S2) is substantially higher than that of the beam using steel plates (COC13S1). The energy dissipation in COC13S1 is 32% of COC13S2 on average, as shown in Figure 12 and Table 2. A comparison with the control (CS), the energy dissipation is (5887 kN.mm) for COC13S1 and (18,566 kN.mm) for COC13S2, which are 73% and 131% of CS (8027 kN.mm), respectively. The lower energy dissipation in COC13S1 can be credited to the inherent behavior of the stiffer steel plates compared to the FRP sheets, which reduced deflections and, consequently, the energy dissipation.

### 3.3. Steel Reinforcement Stresses

The stress values were derived from the readings of strain gages. The experimental stress, σ, was derived from the strain, $\varepsilon_{s}$, obtained from the strain gage readings, considering the bilinear curve:

(1) $$\sigma =\left\{\begin{array}{c} \varepsilon_{s}E_{s}\;\;\;\;\;\;\;\;\;\;\;\;\;\;\;\;for\;\varepsilon_{s}\leq \varepsilon_{y}\\ f_{y}+s\;\left(\varepsilon_{s}-\varepsilon_{y}\right)\;\;\;\;\;\;for\;\varepsilon_{s}\geq \varepsilon_{y} \end{array}\right.$$

where $\varepsilon_{y}$ and $\varepsilon_{u}$ are the yield and ultimate strains, respectively, $E_{s}$ is the modulus of elasticity of steel rebars, $f_{y}$ and $f_{u}$ are the yield and ultimate stresses of steel rebars, $s=\frac{\left({f_{u}-f}_{y}\right)}{\left(\varepsilon_{u}-\varepsilon_{y}\right)}$ (=0 for web rebars, as the ultimate tensile stress is close to the yield stress). According to Table 3, some strain gages were damaged, resulting in missing data. The steel stress measurements for the main longitudinal reinforcement indicated that yielding stress was not achieved, with the exception of COC13S2. In contrast, the vertical and horizontal web rebars close to the openings have yielded.

It is seen from Table 3 that the stresses in the longitudinal rebars of the strengthened beam using the second scheme (Hybrid of FRP sheets and steel plates) are higher than those in first scheme (steel plates alone). This can be attributed to the lower stiffness of FRP laminates used in the second scheme, which results in the transfer of more load to the steel reinforcement. In contrast, the stiffer steel plates in S1 carry a larger portion of the load, resulting in lower stresses in longitudinal rebars. Additionally, the S1 configuration was more conservative. Consequently, despite lower rebar stresses, the conservative S1 design achieved the highest load-carrying capacity, as seen in Figure 11. However, the vertical as well as the horizontal web rebars near the cutouts reached the yield stress. Another observation is that the stresses in vertical web rebars are higher than those in horizontal rebars. The lower stress values in the horizontal stirrups are a result of their positions being slightly offset from the areas of higher stress concentrations.

### 3.4. Ductility

Ductility in RC members is a characteristic that demonstrates their ability to absorb and dissipate energy through inelastic deformation before experiencing failure. It indicates the extent to which the members can undergo inelastic deformation. It is crucial for avoiding sudden failure to ensure the safety of structures.

Different methods are employed to calculate the ductility of RC beams, each offering varying levels of complexity and accuracy. Although displacement and rotation-based ductility are favored for members exhibiting predominant flexural behavior, these methods cannot be employed for RC deep beams, which are shear critical. Even though the failure of deep beams is generally brittle, under certain conditions, these beams can show a reasonable level of ductility. However, the approaches mainly relying on the yielding of tensile reinforcing bars cannot be used for concrete deep beams due to their brittle failure in shear.

Due to the brittle nature of failure of deep beams, some researchers, such as Rao et al. [26], estimated the shear ductility, μ, as the ratio of the energy absorbed up to the ultimate state, $E_{u}$, to the energy absorbed up to the peak load, $E_{p}$:

(2) $$\mu =\frac{E_{u}}{E_{p}}$$

**Figure 13:**
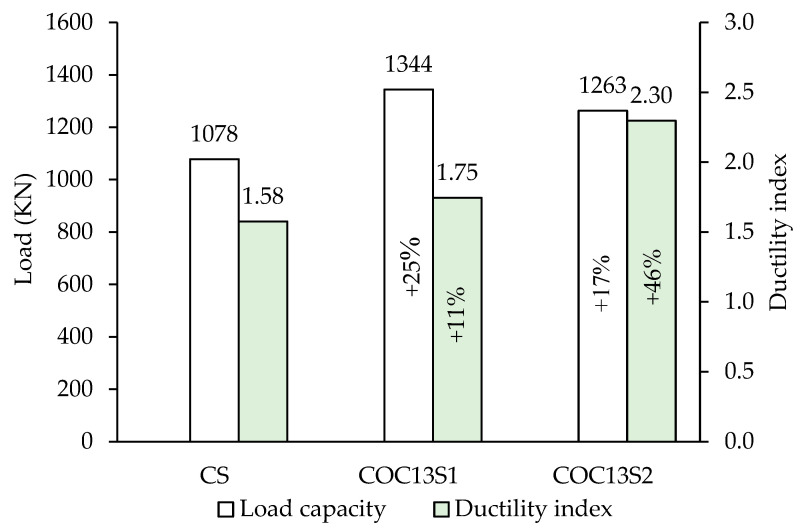
Load capacity and ductility index of tested beams.

The above equation has been adopted in the present study for estimating the shear ductility index. The ultimate state was taken as the state corresponding to a 20% drop in peak load [25]. Figure 13 presents a bar chart showing a comparison of the ductility indices of the tested beams. The ductility index was calculated for the reference and the strengthened beams, which were 1.58, 1.75, and 2.30 for CS, COC13S1, and COC13S2, respectively. The ductility indices of control as well as the strengthened beams calculated vary from 1.58 to 2.3, which are low due to the shear failure of the beams. Both strengthened beams are found to have either close or higher ductility than the control beam (CS). A comparison of the ductility of two strengthening schemes shows that the ductility index of the second scheme, employing FRP composites (COC13S2), is higher than the first scheme, employing steel plates (COC13S1). The relatively lower ductility of the first scheme can be credited to the higher stiffness of steel plates. The ductility index of COC13S1 was 77% of COC13S2 on average.

## 4. Conclusions

Based on the experimental investigation of strengthened continuous high-strength concrete (HSC) deep beams with circular web openings positioned in both interior and exterior strut regions across different spans, two strengthening schemes were evaluated: (a) external and internal steel plates, and (b) a hybrid system combining externally bonded FRP composites with bolted steel plates. By comparing these strengthened configurations with both the control solid beam and unstrengthened beams containing web openings, the following conclusions were drawn:

- In experiments, both the strengthening schemes of using steel plates or FRP composite demonstrated their effectiveness in restoring the strength of deep beams having openings.
- For the beam having circular openings strengthened using steel plates (COC13S1), the load capacity reached 125% compared to the reference solid beam, resulting in a remarkable 110% increase in beam strength compared to the unstrengthened specimen COC13. Similarly, for the beam strengthened using a hybrid FRP/steel plates system (COC13S2), the load capacity reached 117% compared to the reference solid beam, resulting in a load enhancement by 98% compared to the beam with circular openings COC13.
- The energy dissipation in beams retrofitted with FRP composite materials is greater than the beams retrofitted with steel plates. On average, the energy dissipation and ductility index in COC13S1 were 32% and 77% of COC13S2, respectively. These findings suggest that the use of FRP composite materials for strengthening leads to increased energy dissipation, indicating improved structural performance of this scheme of retrofitting.
- Strengthened specimens with steel plates displayed lower deflections due to their higher stiffness compared to specimens strengthened with FRP composite materials. The average mid-span deflection of the beams strengthened using steel plates was almost 52% of the deflection of the beams strengthened using FRP composites. A comparison with the control beam (no openings) shows that the average mid-span deflection of beams strengthened using steel plates is 67% of the control, whereas for the beams strengthened using FRP, it is 31% more than the control. This suggests that the FRP composite material had a more flexible behavior compared to the steel plates.

## Figures and Tables

**Table 1:** Test matrix of two-span continuous deep beams.

Beam ID	Beam Layout
CS	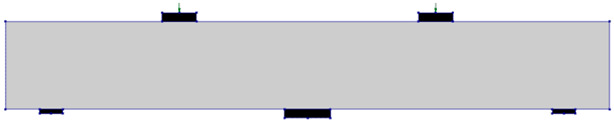
COC13	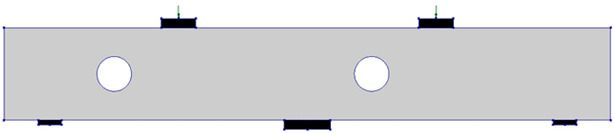
COC13S1	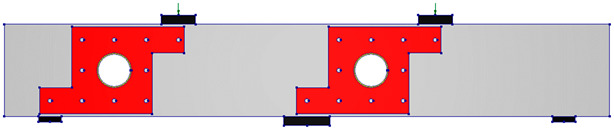
COC13S2	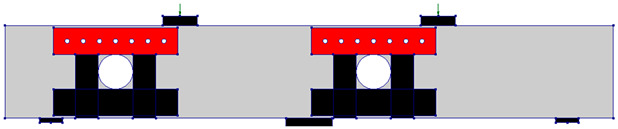

**Table 2:** Comparison of key experimental results of load-deflection response for tested beams.

Beam ID	Total Peak Load (kN)	Vertical Mid-Span Deflection of Left Span (mm)		Vertical Mid-Span Deflection of Right Span (mm)		Energy Dissipated Up to the Ultimate Stage (kN.mm)
		At Peak Load	At Ultimate Stage	At Peak Load	At Ultimate Stage	
CS	1078	5.2	7.0	8.3	12.8	8027
COC13	639	3.0	9.1	4.1	9.9	-
COC13S1	1344	4.5	7.0	4.6	6.4	5887
COC13S2	1263	8.3	17.0	9.1	18.7	18,566

**Table 3:** Comparison of rebar stresses of beams at peak load.

Beam ID	Peak Stress (MPa) in			
	Bottom Rebars	Top Rebars	Horizontal Stirrups	Vertical Stirrups *
CS	466	NA	358	527
COC13	254	93	496	430
COC13S1	441	216	NA	527
COC13S2	560	301	411	527

* The underlined values indicate rebar yielding.

## Data Availability

All data and models generated or used during this study appear in the article.
